# A novel prognostic models for identifying the risk of hepatocellular carcinoma based on epithelial-mesenchymal transition-associated genes

**DOI:** 10.1080/21655979.2020.1822715

**Published:** 2020-09-20

**Authors:** Chen Xiong, Guifu Wang, Dousheng Bai

**Affiliations:** a Dalian Medical University, Dalian, P.R. China; b Department of Hepatobiliary Surgery, Clinical Medical College, Yangzhou University, Yangzhou, P.R. China

**Keywords:** Hepatocellular carcinoma, epithelial-mesenchymal transition, prognosis, nomogram

## Abstract

Several epithelial-mesenchymal transition (EMT)-associated genes (EAGs) have been confirmed to correlate with the prognosis of hepatocellular carcinoma (HCC) patients. Herein, we explored the value of EAGs in the prognosis of HCC relying on data from The Cancer Genome Atlas (TCGA) database. A total of 200 EMT-associated genes were downloaded from the Gene set enrichment analysis (GSEA) website. Moreover, 96 differentially expressed EAGs were identified. Using Gene Ontology (GO) enrichment analysis and Kyoto Encyclopedia of Genes and Genomes (KEGG) pathway analysis, we forecasted the potential molecular mechanisms of EAGs. To identify prognostic EAGs, Cox regression was used in developing a prognostic risk model. Then, the Kaplan-Meier and receiver operating characteristic (ROC) curves were plotted to validate the prognostic significance of the model. A total of 5 prognostic correlated EAGs (P3H1, SPP1, MMP1, LGALS1, and ITGB5) were screened via Cox regression, which provided the basis for developing a novel prognostic risk model. Based on the risk model, patients were subdivided into high-risk and low-risk groups. The overall survival of the low-risk group was better compared to the high-risk group (P < 0.00001). The ROC curve of the risk model showed a higher AUC (Area under Curve) (AUC = 0.723) compared to other clinical features (AUC ≤ 0.511). A nomogram based on this model was constructed to predict the 1-year, 2-year, and 3-year overall survival rates (OS) of patients. Conclusively, we developed a novel HCC prognostic risk model based on the expression of EAGs, which help advance the prognostic management of HCC patients.

**Abbreviations:** HCC: hepatocellular carcinoma; TCGA: The Cancer Genome Atlas; EMT: epithelial-mesenchymal transition; EAGs: EMT-associated genes; GSEA: gene set enrichment analysis; GO: Gene Ontology; KEGG: Kyoto Encyclopedia of Genes and Genomes; PPI: protein-protein interaction; TF: transcription factor; ROC: receiver operating characteristic; K-M: Kaplan-Meier; AUC: the area under the ROC curve; FDR: false discovery rate; TNM: Tumor size/lymph nodes/distance metastasis

## GRAPHICAL ABSTRACT

**Figure:**
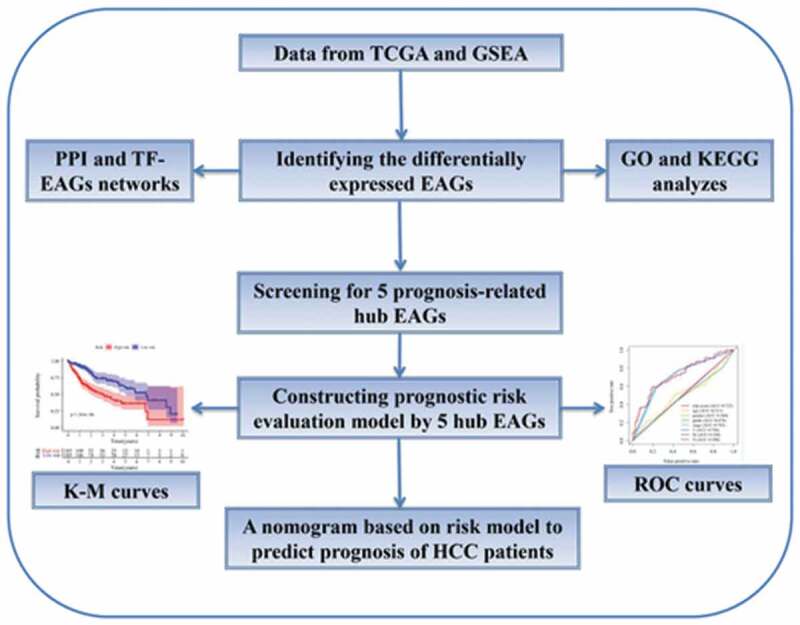

## Introduction

Hepatocellular carcinoma (HCC) is the sixth prevalent cancer and fourth cause of cancer-related mortalities globally [1]. Whilst acknowledging the increasing progress in the diagnosis and treatment of HCC, it does not correspond to its prognosis owing to high metastasis and recurrence rates [2,3]. Notably, HCC patients with similar clinicopathological features exhibit different outcomes and prognosis. Therefore, there is an urgent need to explore the potential metastatic mechanisms in HCC and identify a novel prognosis-related biomarker geared towards advancing the therapy and prognosis of HCC patients.

Metastasis is highly associated with cancer-related morbidity and mortality in HCC [4]. Early metastasis has been reported as a critical factor for recurrence and mortality of HCC, however, the potential mechanisms are still elusive [5]. The Epithelial-mesenchymal transition (EMT) has been revealed to regulate metastasis in HCC [6,7]. For instance, in our previous study, we demonstrated that RING finger protein 38 (RNF38) potentially promotes cancer invasion and metastasis by inducing EMT in HCC [8]. By facilitating transforming growth factor-β (TGF-β) signaling, RNF38 could induce HCC cell EMT, which promoted the HCC cell invasion and metastasis in vitro and in vivo. During the EMT process, the epithelial tumor cells might lose cell-cell contacts by the ablation of E-cadherin, thereby acquiring an increased capacity for migration, invasion, and spread to surrounding or distant tissues. This is critical during the early stages of HCC metastasis [5,9]. Research evidence has discovered several EMT-associated genes (EAGs) used to predict the prognosis of HCC [10-12]. With the advent of microarray and sequencing technologies, several patient genome databases have been constructed. Furthermore, several studies have utilized corresponding genome databases to identify EAGs with a prognosis-predictive value between tumor tissues and normal tissues, consequently establishing a prognosis model for predicting numerous cancers [13,14]. Nonetheless, the prognosis-predictive value of EAGs in HCC is largely underexplored.

In this study, we relied on the mRNA sequencing data and clinical prognostic information of HCC patients downloaded from The Cancer Genome Atlas (TCGA) database to identify prognosis-related EAGs and elicited a prognosis-predictive formula. Using known expression levels of these EAGs and the formula, a risk score of each HCC patient was calculated and used in predicting and evaluating the prognosis of HCC patients.

## Materials and methods

### Data and information acquisition

The total mRNA sequencing data for HCC patients and their corresponding clinicopathological information were downloaded from the TCGA database (April 28, 2020) (https://portal.gdc.cancer.gov/). A total of 200 EAGs were retrieved via the “HALLMARK_EPITHELIAL_MESENCHYMAL_TRANSITION” gene set in the Gene set enrichment analysis (GSEA) (http://www.broadinstitute.org/gsea/index.jsp).

### Identifying the differentially expressed EAGs

To identify the differentially expressed EAGs between HCC samples and normal samples, the mRNA sequencing data for 200 EAGs were analyzed using the “limma” package and the Wilcoxon signed-rank test via R software 3.6.2 (version 3.6.2, https://www.r-project.org/), with adjusted P < 0.05 and thresholds of |log_2_FC| > 1. The analysis results were highlighted by heatmap and boxplot, which was generated using the ggpubr and pheatmap packages in R software.

### Enrichment analysis of differentially expressed EAGs

The potential tumor-associated molecular mechanisms of EAGs was assessed using the Gene Ontology (GO) enrichment analysis and Kyoto Encyclopedia of Genes and Genomes (KEGG) pathway analysis. The GO and KEGG enrichment analysis results were highlighted by a circle plot and a bar plot using the packages in R, which contained “ggpolt2, clusterProfiler, enrichplot, DOSE, etc.”

### Establishing a PPI network and TF-EAGs network

Protein-protein interaction (PPI) network analysis through STRING (STRING: http://www.string-db.org/) was conducted to assess the potential interactions between the proteins coding for the identified differentially expressed EAGs. The PPI network results were visualized using the Cytoscape software (https://cytoscape.org/). Besides, the upstream transcription factor (TF) of differentially expressed EAGs was explored via DAVID (http://david.abcc.ncifcrf.gov/).

### Constructing genetic risk evaluation model according to EAGs

To identify the prognosis-related EAGs in HCC, the univariate Cox and multivariate Cox regression analyses were performed to screen EAGs and determine the corresponding regression coefficient. The cBioPortal database (http://cbioportal.org) was utilized to analyze the mutations of these EAGs in HCC patients. Following the results of multivariate Cox regression analyses, a prognosis-predictive formula was generated: Risk score=expression of gene 1×G1+expression of gene 2×G2+⋯+expression of gene n×Gn, where Gn represents the prognostic gene. Then, a risk evaluation model of EAGs was established using R software via the package “glmnet”. Kaplan-Meier (K-M) curve, the area under curve (AUC) of the receiver operating characteristic (ROC) curve, and the KEGG enrichment analyses were performed to verify the predictive value of the risk evaluation model. Eventually, a predictive nomogram was constructed using the package “rms” in R to predict the prognosis and survival rate of patients.

### Statistical analysis

P-value < 0.05 was considered statistically significant. The data for total mRNA sequencing were normalized using log2 transformation. The R software 3.6.2 (https://www.r-project.org/) and Perl languages (https://www.perl.org/) were used for all statistical analyses.

## Results

### Identifying differentially expressed ARGs

First, mRNA-seq data for HCC patients and their clinical information were downloaded from the TCGA database. The mRNA-seq data for 56,536 genes were obtained from 374 HCC samples and 50 normal samples from the TCGA database. The clinicopathological information including age, gender, histological grade, TNM stage, T stage, N stage, and M stage were obtained for 371 HCC patients. Subsequently, 200 EAGs from the “HALLMARK_EPITHELIAL_MESENCHYMAL_TRANSITION” gene set in GSEA were identified, which were listed in Supplementary Table 1. It provided a list of 200 genes which have been demonstrated to be associated with EMT.

**Figure 1.**
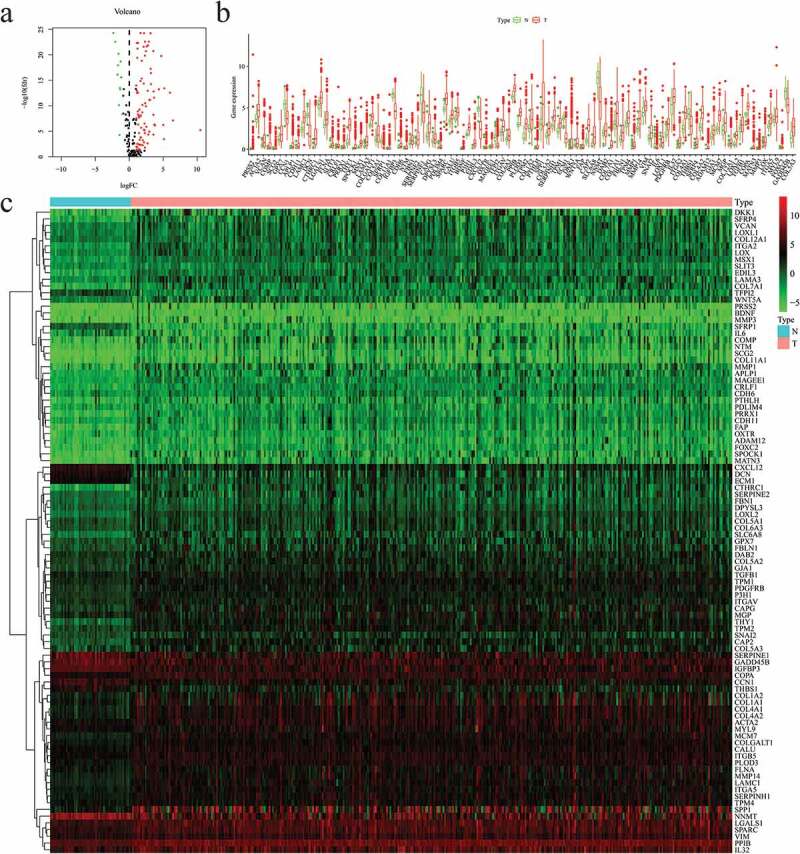
Differentially expressed 96 EMT-associated genes (EAGs) in hepatocellular carcinoma (HCC) and non-tumor hepatic samples. (a) The volcano map of 96 screened EAGs. (b) The expression of 96 screened EAGs in a boxplot. (c) The expression of 96 screened EAGs in a heatmap. EMT: Epithelial-mesenchymal-transition.

Thereafter, the mRNA expression data and screened for differentially-expressed EAGs between HCC samples and normal samples were analyzed using the package “limma” and the Wilcoxon signed-rank test in R (FDR < 0.05, ∣logFC∣ > 1). Based on the results, 12 downregulated EAGs and 84 upregulated EAGs were screened from HCC samples, which were showed in volcano plot (Figure 1(a)). The expression levels of these genes in HCC samples and normal samples were visualized via boxplot and heatmap (Figure 1(b-c)).

### GO enrichment and KEGG pathway analyses

**Figure 2.**
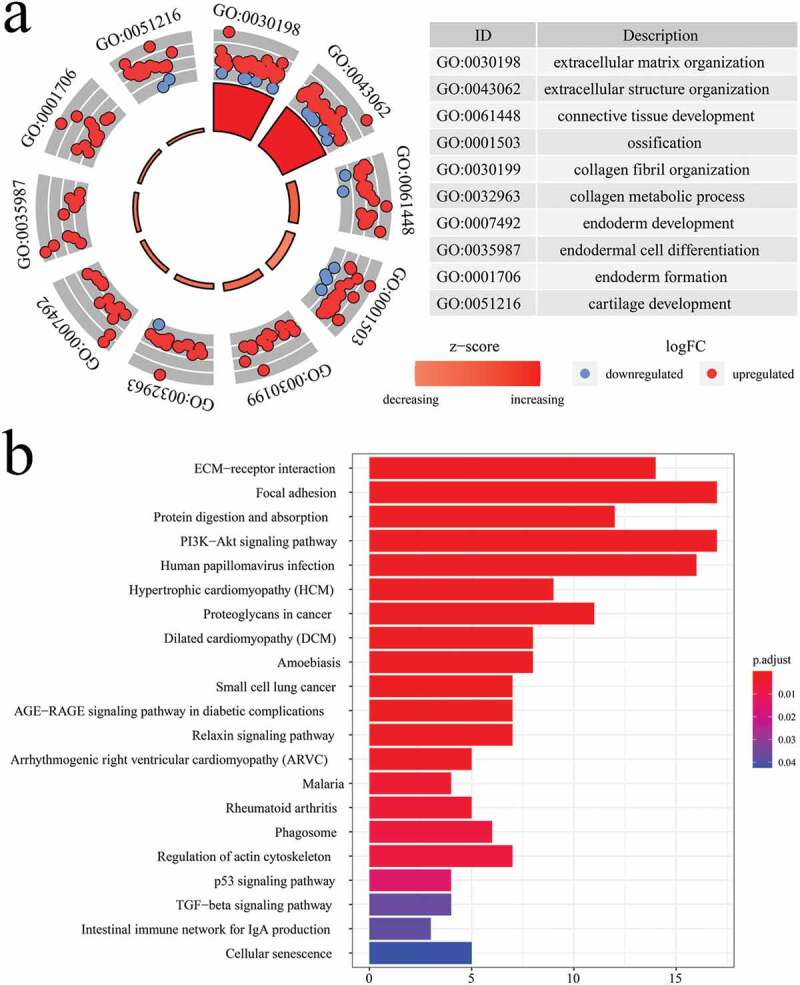
GO enrichment analysis and KEGG pathway analysis of 96 screened EAGs.

GO and KEGG analyses were conducted to explore the potential molecular mechanism and biological functions of the 96 identified EAGs. Based on the GO analysis results, the EAGs were primarily implicated in the biological process associated with the development of tissue or embryo (Figure 2(a)). Besides, the KEGG analysis results revealed that the 96 EAGs were highly enriched in pathways for ECM receptor interaction, focal adhesion, proteoglycans in cancer, and the oncogenic PI3K/AKT signaling (Figure 2(b)).

(a) Circle plot of enriched GO terms. (b) Bar plot of the enriched pathways. GO: Gene ontology; KEGG: Kyoto Encyclopedia of Genes and Genomes.

### PPI network and TF-EAGs network

**Figure 3.**
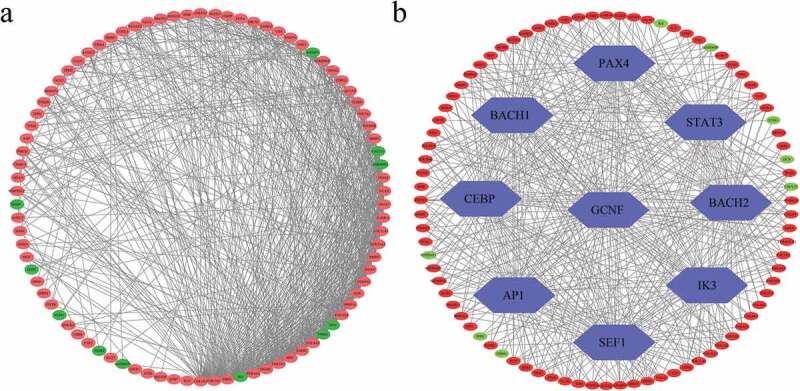
(a) PPI networks among 96 screened EAGs. (b) Upstream TF-EAGs regulatory networks. Green nodes represent down-regulated genes, while red nodes represent up-regulated genes. PPI: protein-protein interaction; TF: transcription factor.

The STRING online website was used to analyze the potential interactions between the proteins coded by the EAGs and visualized the results using the Cytoscape software (Figure 3(a)). The Cytoscape software depicted the detailed protein relationships. Also, the DAVID website was used to predict upstream common transcription factors of the identified EAGs. Following the TF prediction results, the major common transcription factors of the EAGs included PAX4, STAT3, BACH2, IK3, SEF1, AP1, CEBP, BACH1, and GCNF (Figure 3(b)).

### Screening for prognostic-related hub EAGs

**Figure 4.**
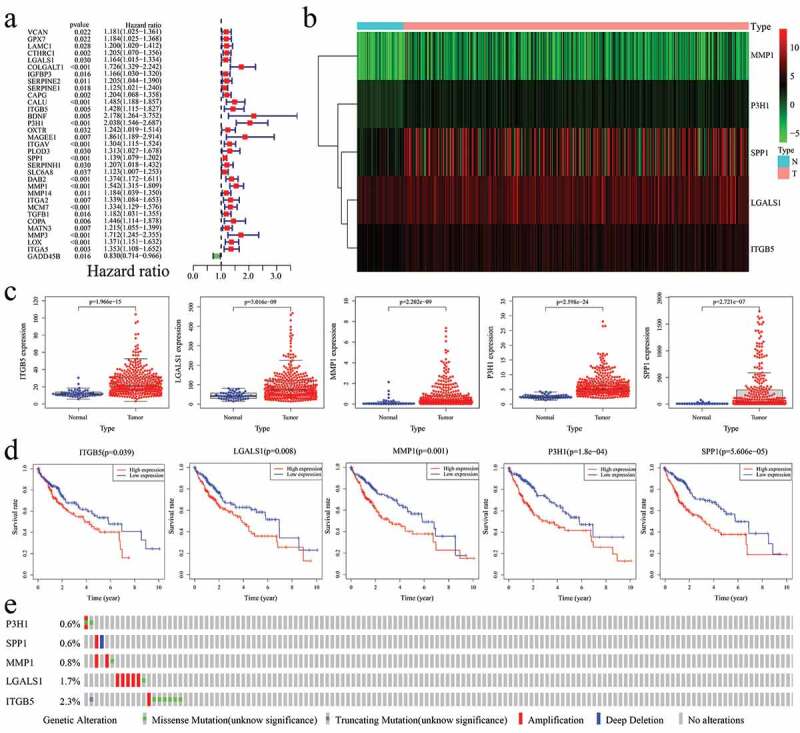
Identification of prognosis-related EAGs in HCC. (a) Univariate Cox regression results of prognosis-related EAGs in HCC. (b) Heatmap for the 5 identified prognosis-related EAGs after multivariate Cox regression. (c) Expressions of 5 identified EAGs between HCC and normal samples. (d) Overall survival rates in K-M curves to verify the prognostic value of 5 EAGs in HCC. (e) The mutations of 5 EAGs in 353 HCC samples in the cBioPortal database.

After identifying the differentially expressed EAGs, the univariate Cox regression analysis was performed to screen for prognostic-related EAGs. In the corresponding results, 32 EAGs were positively correlated to prognosis (hazard ratio > 1), whereas 1 EAG were negatively correlated to prognosis (0 < hazard ratio < 1) (Figure 4(a)). Subsequently, the multivariate cox regression analysis was adopted to identify the prognostic-related hub EAGs and obtain their regression coefficients (Table 1). The results were displayed a heatmap, which showed the expression levels of P3H1, SPP1, MMP1, LGALS1, and ITGB5 in tumor tissues and normal tissues (Figure 4(b)). Further, the correlation between these 5 genes and the prognosis of HCC patients was analyzed. First, the expressions of these 5 genes in HCC and normal tissues were determined. Unlike the expression in normal tissue, all the 5 genes were significantly and highly expressed in HCC tissue (Figure 4(c)). Then, the K-M curve was used to verify the difference in survival rates between highly-expressed and lowly-expressed groups of the 5 hub EAGs. Notably, the higher expression of 5 EAGs implied a lower survival rate of HCC patients (Figure 4(d)).

**Table 1.** Multivariate Cox regression results of prognosis-related EAGs in HCC.

Gene id	Coefficient	HR	HR.95L	HR.95H	P value
P3H1	0.488729	1.630243	1.196957	2.220374	0.001932
SPP1	0.106081	1.111912	1.044272	1.183933	0.000924
MMP1	0.273528	1.314595	1.077784	1.603437	0.006952
LGALS1	−0.16391	0.848822	0.710058	1.014705	0.071911
ITGB5	0.215136	1.24003	0.93037	1.652757	0.142209

EAGs, epithelial-mesenchymal transition-associated genes; HCC, hepatocellular carcinoma. HR, hazard ratio.

Furthermore, the cBioPortal database (http://cbioportal.org) was used to investigate the mutation conditions of 5 hub ERGs. According to the data for 353 HCC patients from the database, 21 patients had several different mutations of 5 hub ERGs (Figure 4(e)). From the results, it was found that 0.57% patients had missense mutations whereas 0.28% patients had amplifications in P3H1; 0.28% patients had amplifications while 0.28% patients had deep deletions in SPP1; 0.28% patients had missense mutations while 0.56% patients had amplifications in MMP1; 0.28% had missense mutations while 1.41% had amplifications in LGALS1; 1.7% patients had missense mutations, 0.28% patients had amplifications, and 0.28% patients had truncating mutations in ITGB5.

### Constructing the EAGs-related prognosis risk model

**Figure 5.**
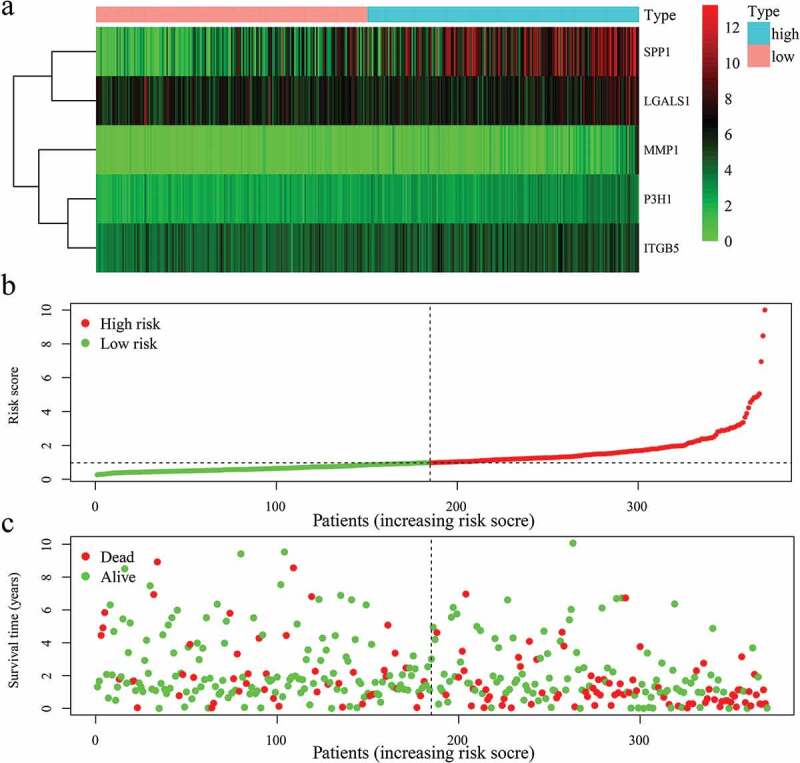
The risk score constructed by 5 EAGs to predict OS in HCC patients. (a) Heatmap of 5 EAGs’ expression profile between high and low-risk groups. (b) The risk score distribution of HCC patients. (c) Correlations between survival times and the survival status of HCC patients. OS: overall survival rates.

**Figure 6.**
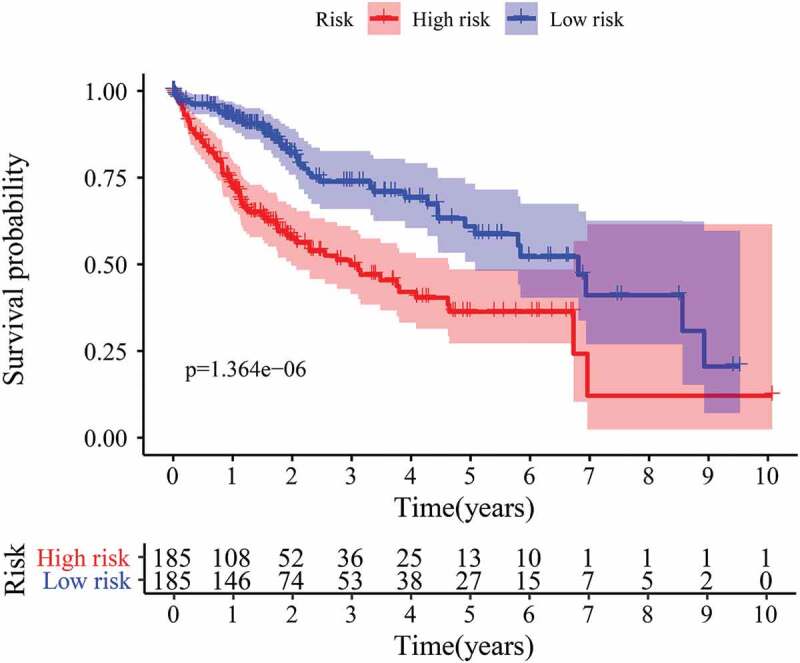
K-M curve for HCC patients in high/low-risk group.

Upon obtaining the list of differentially expressed EAGs, a prognosis prediction model of HCC patients was developed using the screened EAGs and their regression coefficients. The formula used: EAGs-related prognosis risk model = (0.488729 * P3H1) + (0.106081 * SPP1) + (0.273528 * MMP1) – (0.16391 * LGALS1) + (0.215136 * ITGB5), from which the risk value of each patient was calculated. Patients were ranked in ascending order of the risk value and categorized into a high-risk group and a low-risk group through a comparison of the risk value with the median risk value (Figure 5(b)). The expression levels of 5 hub EAGs between high-risk group and low-risk group were plotted in Figure 5(a). The survival time of patients is shown in a scatterplot (Figure 5(c)), which revealed a significantly shorter survival time of patients in the high-risk group compared to those in the low-risk group. The K-M curve and log-rank methods demonstrated the correlations between the risk model and the prognosis of patients. Notably, the survival probability of patients in the low-risk group was remarkably higher than the survival probability of patients in the high-risk group (P < 0.00001) (Figure 6).

All regression coefficients of 5 EAGs were positive except for LGALS1 which showed a negative regression coefficient, an implication that the high expression of LGALS1 might be a positive correlation with the prognosis of the HCC patients. However, all the 5 screened EAGs had a negative correlation between their high expressions and survival rate of patients by the K-M curve (Figure 4(d)). Besides, the 5 EAGs were significantly and highly expressed in HCC tissues than in normal tissues (Figure 4(c)).

### Association between the risk model and clinical features

**Figure 7.**
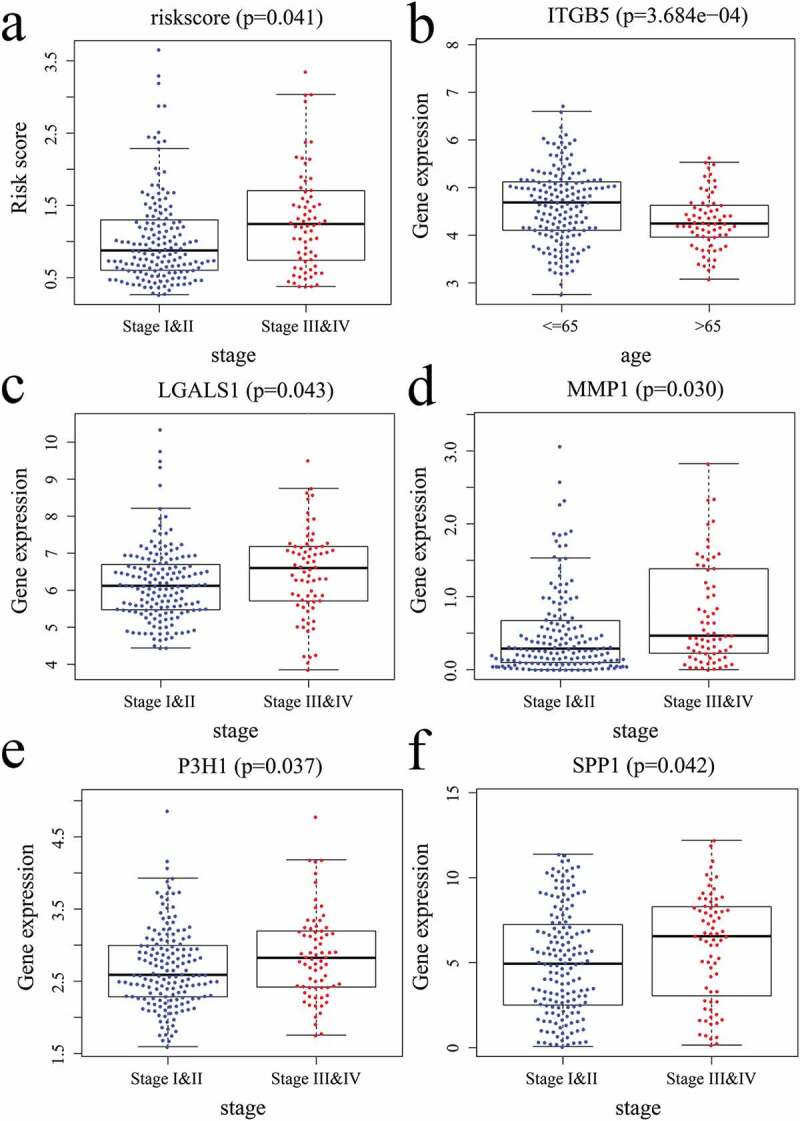
Correlations between the risk score/5 EAGs and patient’s clinical features. (a) risk score and pathology stage. (b) ITGB5 expression level and age. (c) LGALS1 expression level and pathology stage. (d) MMP1 expression level and pathology stage. (e) P3H1 expression level and pathology stage. (f) SPP1 expression level and pathology stage.

The relationship between the risk model/5 EAGs and the clinical characteristics of patients were analyzed based on the gene expression data and clinicopathologic features. Of note, the risk score was revealed to be associated with the TNM stage (Figure 7(a)); ITGB5 was correlated with age (Figure 7(b)); LGALS1, MMP1, P3H1, and SPP1 were associated with TNM stage (Figure 7(c-f)).

### Comparing the independent predictive value of the risk model with clinical features

**Figure 8.**
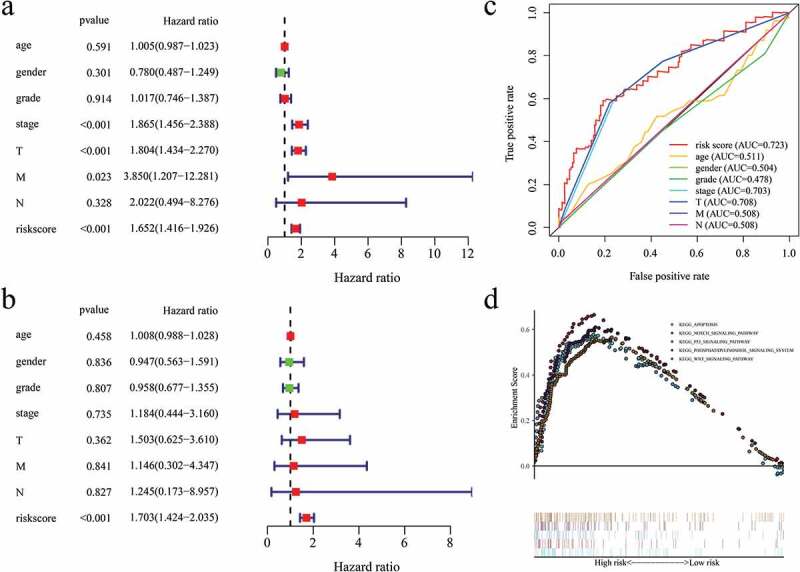
(a) Univariate Cox regression analysis of risk score in HCC. (b) Multivariate Cox regression analysis of risk score in HCC. (c) ROC curves comparing the prognosis predictive value of the risk model and different clinical features in HCC. (d) KEGG pathway analysis of 5 identified EAGs.

A comparison of the independent predictive value of the risk model with the clinical features was conducted through the univariate Cox and multivariate Cox regression analyses (Figure 8(a-b)). The result revealed that the risk score in our model was the only statistically significant independent prognostic predictor (P < 0.001, Hazard ratio >1). Besides, the ROC curves and corresponding AUCs were used to compare the prognosis predictive value of the risk model and the different clinical features (Figure 8(c)), where the AUC of the risk model was 0.723. Based on these findings, the risk model had the highest predictive value compared to other clinical features, suggesting the highest prognostic predictive power.

### KEGG analysis of the hub EAGs

The KEGG enrichment analysis was performed to evaluate the potential mechanism associated with the 5 hub EAGs in HCC (Figure 8(d)). In this figure, results revealed that the 5 EAGs were highly enriched in 5 biological pathways, including apoptotic pathway, notch signaling pathway, P53 signaling pathway, phosphatidylinositol signaling system pathway, and WNT signaling pathway.

### Establishing a prognosis predictive nomogram

**Figure 9.**
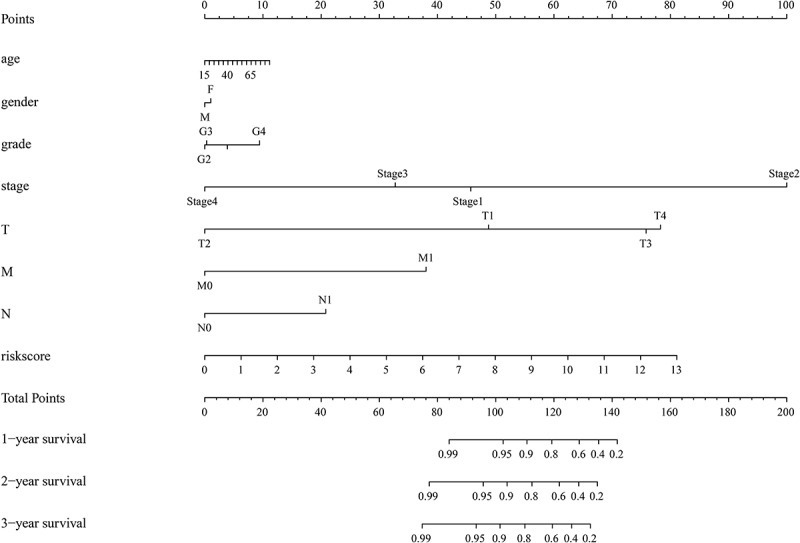
A nomogram predicting the 1-, 2-, and 3- year overall survival of HCC patients.

A prognostic nomogram based on the risk model was developed for the clinical application of our risk model (Figure 9). Based on the risk score and clinical features of the patients, a total point was calculated to predict the 1-year, 2-year, and 3-year overall survival rates (OS) of HCC patients.

## Discussion

Hepatocellular carcinoma HCC is a life-threatening malignancy characterized by a high recurrence rate and poor prognosis [15,16]. Metastasis, a prevalent phenotype in the majority of cancers, is primarily associated with worse prognosis and death of HCC patients, where EMT is a crucial step [6,17]. Reports indicate that the management of HCC patients requires an early prediction of prognosis [18]. Previously, the clinicopathological features of patients with HCC were used to predict their prognosis. However, recent studies have shown that clinicopathological features are inadequate to make a precise prognostic assessment [19,20]. It has been demonstrated that the EAGs have the predictive potential in the prognosis of some tumors [13,14]. Therefore, there is a need to explore an advanced strategy based on the EMT-associated genes for the prognostic assessment of HCC patients.

Herein, we constructed a prognostic-predictive model using the total gene expression level of EAGs based on the TCGA database geared towards elucidating the prognostic-predictive value of overall EAGs. First, based on the data from the TCGA database and a 200 EAGs list from the GSEA, 12 downregulated EAGs and 84 upregulated EAGs in HCC samples were screened. GO enrichment and KEGG analyses were performed to reveal potential biological processes and pathways correlated with these EAGs. A PPI network analysis network was performed by the STRING, which showed the potential interactions between the coding proteins of 96 EAGs. Besides, we executed a TF-EAGs network to investigate the possible transcription factors of these 96 EAGs. Among these transcription factors (TFs), CEBP, AP1, STAT3 have been reported to mediate oncogenic effects of other substances in HCC, indicating that these TFs might have potential research values in HCC [21-23]. Through the analysis of gene sequencing data and clinical prognostic information of HCC patients, a prognostic risk model was constructed by 5 hub prognosis-related EAGs, including LGALS1, MMP1, P3H1, ITGB5, and SPP1. The results of univariate and multivariate regression analysis showed that the risk model might be regarded as an independent prognostic model that can accurately evaluate the overall survival in HCC (P < 0.001, Hazard ratio > 1). K-M curve and ROC curve have been utilized to illustrate the predictive value of this risk model. The K-M curves showed that the overall survival of the low-risk group was remarkablely better compared to the high-risk group (P < 0.00001). Besides, the AUC value of risk model was higher than any other clinical features (AUC=0.723). All results showed that this model has a higher prognostic predictive value than other clinical features of HCC patients. Finally, we created a nomogram based on the risk score and clinical features of patients geared towards directly predicting the overall survival rates.

Previous investigations have reported that some of these 5 hub EAGs were associated with the development and prognosis of HCC patients [24-26]. For instance, the ITGB5 (Integrin-β5), a member of the integrin family, promotes tumorigenesis in HCC, thus might be a potential independent prognostic biomarker for HBV-related HCC patients [25,27]. Additionally, Matrix metalloproteinase 1 (MMP1), a member of zinc-dependent endoproteases, degrade the extracellular matrix (ECM) by breaking down its structural proteins and has been demonstrated to regulate the EMT process in HCC [28]. Elsewhere, high expression of MMP1 was linked to a poorer prognosis in HCC [29]. Besides, Secreted phosphoprotein 1 (SPP1), an integrin-binding participating in tumorigenesis and metastasis, is over-expressed in numerous cancers, including HCC [30]. Notably, the prognostic-predictive value of SPP1 in HCC has widely been verified and applied in many studies [31-33]. LGALS1, also called galectin-1, was revealed to regulate immune responses, cancer metastasis, and cell survival in a few tumors, such as glioma, leukemia, and oral cancer [34-36]. Nevertheless, so far, the role of LGALS1 in HCC is largely unexplored. Similarly, there is no evidence on the role of P3H1 in HCC. Therefore, we propose in-depth assessments on the role and prognostic value of LGALS1 and P3H1 in predicting HCC. This might aid in the discovery of novel biomarkers for the prognosis of HCC patients.

This study established a reliable prognostic risk model for HCC patients using the sequencing data of EAGs and clinical information from the TCGA database. As a result, the prognostic-predictive function of our risk model was highly satisfactory compared to other clinicopathological features, which were illustrated by K-M curves and ROC curves. Notably, this is a maiden study that incorporated the prognosis of HCC patients with expression of their EMT-associated genes. This might provide a novel method for the high and precise prognosis of HCC as well as evaluate the survival of HCC patients.

## Conclusion

In conclusion, we developed a 5-gene prognostic risk model based on the EMT and established a nomogram for HCC. This provides a new method for predicting the survival and prognosis of HCC patients using the expressions of 5 EAGs and clinicopathological information. Comparing to tradtional predict way via clinical features, a better prognostic prediction and management of HCC patients can be achieved via this risk model, and thereby contributing to a higher survival rate. This is the first study to predict prognosis of HCC patients via the expression levels of EMT-associated genes. Our findings therefore provide novel insights into the relationship between HCC and EMT.

## Supplementary Material

Supplemental Material

## Data Availability

The datasets analyzed was acquired from The Cancer Genome Atlas (TCGA) database (https://portal.gdc.cancer.gov/).

## Highlights

1. The expression levels of EMT-associated genes were related with prognosis of HCC patients.
2. EMT-associated genes were demonstrated to have independent prognosis-predictive values in HCC.
3. EMT-associated genes may be prognostic indicators in HCC.
