# Supplementary material for: A novel prognostic models for identifying the risk of hepatocellular carcinoma based on epithelial-mesenchymal transition-associated genes
Source: Bioengineered. 2020 Sep 20;11(1):1034–46. doi: 10.1080/21655979.2020.1822715 (PMC8291854; doi:10.1080/21655979.2020.1822715)
Supplement: Supplemental Material [file KBIE_A_1822715_SM8100.docx]

Table 1. Multivariate Cox regression results of prognosis-related EAGs in HCC.

| Gene id | Coefficient | HR | HR.95L | HR.95H | P value |
| --- | --- | --- | --- | --- | --- |
| P3H1 | 0.488729 | 1.630243 | 1.196957 | 2.220374 | 0.001932 |
| SPP1 | 0.106081 | 1.111912 | 1.044272 | 1.183933 | 0.000924 |
| MMP1 | 0.273528 | 1.314595 | 1.077784 | 1.603437 | 0.006952 |
| LGALS1 | -0.16391 | 0.848822 | 0.710058 | 1.014705 | 0.071911 |
| ITGB5 | 0.215136 | 1.24003 | 0.93037 | 1.652757 | 0.142209 |
| EAGs, epithelial-mesenchymal transition-associated genes; HCC, hepatocellular carcinoma. HR, hazard ratio | | | | | |
